# Stable Quantitative Resistance Loci to Blackleg Disease in Canola (*Brassica napus* L.) Over Continents

**DOI:** 10.3389/fpls.2018.01622

**Published:** 2018-11-23

**Authors:** Harsh Raman, Rosy Raman, Simon Diffey, Yu Qiu, Brett McVittie, Denise Maria Barbulescu, Phil Anthony Salisbury, Steve Marcroft, Regine Delourme

**Affiliations:** ^1^Graham Centre for Agricultural Innovation, Wagga Wagga Agricultural Institute, Wagga Wagga, NSW, Australia; ^2^Centre for Bioinformatics and Biometrics, University of Wollongong, Wollongong, NSW, Australia; ^3^Agriculture Victoria, Grains Innovation Park, Horsham, VIC, Australia; ^4^Faculty of Veterinary and Agricultural Sciences, The University of Melbourne, Melbourne, VIC, Australia; ^5^Marcroft Grains Pathology, Horsham, VIC, Australia; ^6^IGEPP, INRA, Agrocampus Ouest, Université Rennes, Le Rheu, France

**Keywords:** blackleg, *Leptosphaeria maculans*, quantitative resistance, ascospore shower, genetic mapping, physical mapping

## Abstract

The hemibiotrophic fungus, *Leptosphaeria maculans* is the most devastating pathogen, causing blackleg disease in canola (*Brassica napus* L). To study the genomic regions involved in quantitative resistance (QR), 259–276 DH lines from Darmor-*bzh*/Yudal (DYDH) population were assessed for resistance to blackleg under shade house and field conditions across 3 years. In different experiments, the broad sense heritability varied from 43 to 95%. A total of 27 significant quantitative trait loci (QTL) for QR were detected on 12 chromosomes and explained between 2.14 and 10.13% of the genotypic variance. Of the significant QTL, at least seven were repeatedly detected across different experiments on chromosomes A02, A07, A09, A10, C01, and C09. Resistance alleles were mainly contributed by ‘Darmor-*bzh*’ but ‘Yudal’ also contributed few of them. Our results suggest that plant maturity and plant height may have a pleiotropic effect on QR in our conditions. We confirmed that *Rlm9* which is present in ‘Darmor-*bzh*’ is not effective to confer resistance in our Australian field conditions. Comparative mapping showed that several *R* genes coding for nucleotide-binding leucine-rich repeat (LRR) receptors map in close proximity (within 200 Kb) of the significant trait-marker associations on the reference ‘Darmor-*bzh*’ genome assembly. More importantly, eight significant QTL regions were detected across diverse growing environments: Australia, France, and United Kingdom. These stable QTL identified herein can be utilized for enhancing QR in elite canola germplasm via marker- assisted or genomic selection strategies.

## Introduction

Canola (rapeseed, *Brassica napus* L.) is one of the major oilseed crops of the world, contributing 13% of oil supply. In recent years, high market price of canola, plasticity in flowering time window, and response to moisture supply (rainfall and irrigation) has encouraged farmers to grow this crop in narrow rotations across a range of environments. Blackleg disease, caused by the hemibiotrophic fungal pathogen, *Leptosphaeria maculans* undermines sustainable production of canola especially in Australia, Canada, France, and United Kingdom (West et al., 2001; Fitt et al., 2006). Yield reductions of up to 80% have been reported (West et al., 2001) in addition to marked reduction in seed and oil quality attributes. Several control measures such as crop rotation, stubble management and use of fungicides, as seed dressing and foliar application, have been used to control the disease (West et al., 2001; Marcroft et al., 2012a,b). However, widespread use of fungicides such as Jockey^^®^^ (Fluquinconazole) for seed dressing, and Impact^^®^^ (Flutriafol) as a fertilizer has led the development of fungicide resistant isolates (Van de Wouw et al., 2017). Breeding for host resistance is considered as an effective and environment friendly strategy to protect various crops, including canola from *L. maculans* infection. It has become a major target trait in Australian canola breeding programs after the severe epidemics of the early 1970s (Salisbury and Wratten, 1999).

There is a wide range of genetic variation in resistance to blackleg among canola cultivars. Two types of resistance, qualitative resistance conferred by race-specific *R* genes, and quantitative resistance (QR) conferred by non-race specific genes (QTL) have been deployed to combat this disease in commercial cultivars. To date, at least fifteen *R* genes have been identified in *B. napus* and its related species, *B. rapa* and *B. juncea* (Raman et al., 2013). Although these *R* genes provide ‘complete’ protection from *L. maculans* infection, this resistance often becomes ineffective, particularly when the frequency of *avr* alleles is high in nature (Sprague et al., 2006; Van de Wouw et al., 2010a,b, 2014). Even deployment of *R* gene pyramids, in cultivars such as Surpass400 and Hyola50 has not provided long-term durability against highly diverse *L. maculans* races in Australia. Research has shown that QR is more ‘durable’ and provides long-term protection to host plants (Delourme et al., 2006a; Brun et al., 2010; Delourme et al., 2014). However, identification of such resistance is very difficult to achieve particularly when *R* gene(s) are present in the same cultivar/accession. Several canola breeding programs rely on screening germplasm under blackleg nurseries across multiple locations and selecting the best standing plants or surviving lines at the end of the growing season. This practice is quite effective but is not highly efficient for making genetic gains by incorporating QR in breeding lines.

Genetic mapping of QR via traditional QTL and association (genome wide association studies) mapping approaches has enabled the identification of quantitative loci for resistance to *L. maculans* on the genetic/physical maps of *B. napus* (Delourme et al., 2008; Kaur et al., 2009; Jestin et al., 2011; Jestin et al., 2012; Raman et al., 2012a,b; Raman R. et al., 2016; Raman H. et al., 2016; Fopa Fomeju et al., 2014; Jestin et al., 2015; Larkan et al., 2016a; Kumar et al., 2018). One of the major limitations of QTL mapping studies in genetic improvement programs has been the stability of QTL across environments and the magnitude of QTL effects in different genetic backgrounds (Pilet et al., 2001; Delourme et al., 2008; Jestin et al., 2012; Huang et al., 2016; Larkan et al., 2016a; Kumar et al., 2018). Of the various genetic analyses/QTL identification studies conducted to date, Darmor-*bzh*/Yudal (DY) DH population of *B. napus* has been extensively evaluated for resistance to blackleg in diverse environments across Europe (Pilet et al., 1998; Delourme et al., 2008; Huang et al., 2009; Jestin et al., 2012; Kumar et al., 2018). However, the effectiveness of QR and those QTL identified in Europe have not been tested against highly diverse pathogenic Australian *L. maculans* populations.

In this study, we evaluated the DYDH population under Australian field, glasshouse and shade house conditions using an ascospore shower test and identified QR loci. We further compared the physical locations of QTL that are identified in Australia (this study), with those identified in France and United Kingdom to identify consistent genomic regions involved in resistance across continents. Research findings would be useful to decipher functional genes underlying such regions as well as to provide target regions (molecular markers) for marker-assisted and genomic selection in canola breeding programs.

## Materials and Methods

A doubled haploid (DH) population derived from a cross between ‘Darmor-*bzh*’ and ‘Yudal,’ comprising 276 lines (Foisset et al., 1995, 1996) was accessed from INRA, Le Rheu, France. ‘Darmor-*bzh*’ is a French winter type and double-low oil quality cultivar, while ‘Yudal,’ is a Korean Spring type cultivar. Besides resistance to blackleg disease, this population also segregates for a range of traits such as plant height, flowering time, oil content (Delourme et al., 2006b) and quality, and tolerance to manganese (Mn^2+^) toxicity (Raman et al., 2017). ‘Darmor-*bzh*’ carries the dwarf *Bzh* gene and is known to carry *Rlm9* for qualitative resistance to *L. maculans* (Foisset et al., 1995; Delourme et al., 2004). Seed of parental lines as well DH lines was multiplied in caged tents to produce ‘pure’ seed for phenotypic and genetic analyses.

### Fungal Isolates

Pure cultures of *L. maculans* were obtained from Marcroft Grains Pathology, Horsham (Australia) and then multiplied on V8 agar medium at the Wagga Wagga Agricultural Institute (WWAI). One isolate D10 (PHW1223, *AvrLm5, AvrLm6, AvrLm8, AvrLm9, LepR3;* Marcroft et al., 2012b) was used to screen for expression of resistance conferred by *Rlm9* gene. D13 isolate (having *avrLm9*) was used to reconfirm the resistance mediated by *Rlm9* among resistant lines.

### Phenotyping of DH Lines for Resistance to *L. maculans*

#### *R* Gene Mediated Resistance

To pinpoint whether *Rlm9* gene contributes to resistance under field conditions, we evaluated 259 DH lines of a DYDH population using a single spore isolate of *L. maculans* following randomized complete block design.

*2016 Glasshouse Trial:* We used a randomized complete block design where thirty eight 7 column by 2 row seedling trays were placed on a glasshouse bench in the manner presented in Supplementary Figure 1. Even numbered trays were one replicate block and odd numbered trays the other replicate block. Two hundred and sixty six lines, comprising 259 DH lines and 7 control lines were sown once in each replicate block. The treatment and blocking structure for the 2016 glasshouse trial can be written as

Blocking structure: Block
Treatment structure: Geno

where Block is a factor with 2 levels and Geno is a factor with 266 levels.

Expression of the *R* gene was assessed under controlled environment (CE) conditions at the cotyledon stage. Seven to 10 day old seedlings were inoculated with conidial spore suspension (5 × 10^6^ spores/mL) containing 0.01% of Tween 20 as described previously (Raman et al., 2012b). Both lobes of each cotyledon were punctured with a plastic micropipette tip and then inoculated with 10 μL of spore suspension. Inoculated seedlings were subjected to a humidity chamber to ensure optimum wetness in the CE for 48 h. Trays were then shifted to the CE room and maintained at 20°C ± 1C with 16–8 h photoperiod. The disease symptoms started to appear after 10–12 days from inoculation. Three week after inoculation, all DH lines and other parental lines including ‘checks’ were scored for disease severity based on a scale described previously (Koch et al., 1991). DH lines as well as six parental lines/check cultivars, Darmor, AV-Garnet, and TornadoTT were used as resistant, while Yudal, Westar (no *R* gene), and Caiman were used as susceptible controls.

In order to test whether the same set of *R* gene(s) expressed at the cotyledon stage also control resistance at the adult plant stage, we raised all DH lines that were inoculated with isolate PHW1223 in a glasshouse using the same experimental design as described above. Five months after inoculations, all DH lines were scored for internal infection using a 1–5 scale; where 0 = healthy, 1 = 1–20% stem discoloration (internal infection), 2 = 21 to 40% internal infection, 3 = 41–60% internal infection, 4 = 61–80% internal infection and 5 = 81–100% internal infection, as described in Larkan et al. (2016a).

#### Quantitative Genes Mediated Resistance

Three experiments were carried out in 2014, 2015, and 2016 to assess resistance to *L. maculans* in the DYDH population following statistically valid designs under shade house and field conditions.

##### Ascospore shower test

The 2014 ascospore shower (tub) test using the mixed stubble collected after harvest of canola varieties was conducted as described previously (Marcroft et al., 2012b) in a multi-phase experiment comprising a glasshouse, growth chamber and shade house phase. Seedlings were grown in 20 cell (4 rows by 5 columns) seedling trays for approximately 2 weeks in a glasshouse. In each tray, a single DH line (or a parental line) was allocated to the 4 cells in each tray column, i.e., each tray contained 5 DH lines. Each DH line was replicated twice, i.e., was allocated to a tray column in two different trays. The two parental lines were replicated 30 times. The experimental unit at the glasshouse (and growth chamber) stage of the experiment is a tray column. Trays were then allocated to one of two growth chambers in such a way that each growth chamber contains 4 seedlings, i.e., one experimental unit, of each DH line. The growth chamber phase of the experiment lasts 2 days in which time blackleg fungal spores have an opportunity to cause seedling infection. At the end of the growth chamber phase the 4 single DH line seedlings in a tray are transplanted into a pot which is then placed in a shade house. The shade house is divided into two bays (left and right) with pots arranged in a 4 column by 86 row arrays in one bay and a 4 column by 69 row arrays in the other bay, i.e., a total of 620 pots. A restricted randomization was used to assign growth chamber tray experimental units to non-rectangular blocks of 5 pots in the shade house. Pots were kept in the shade house for approximately 3 weeks and then each plant within a pot was visually assessed for internal infection. Internal infection data was collected on 269 DH lines, the two parental lines and 6 Australian canola varieties: Crusher, CB-Telfer, Hyola444, ThumperTT, ATR-Marlin, ATR-Stingray which were used as resistant and susceptible controls. The treatment and blocking structure for the 2014 tub test trial can be written using the symbolic notation of Wilkinson and Rogers (1973) as

Blocking structure: GC + GC.Tray + GC.GCCol.GCRow
+ SHBay + SHBay.SHBlock
Treatment structure: Geno

where GC indexes growth chambers and is a factor with 2 levels, Tray is a unique identifier for trays within growth chambers, GGCol and GCRow indexes the spatial arrangement of tray experimental units within each growth chamber, SHBay is a factor which indexes the two sides of the shade house and SHBlock indexes non-rectangular blocks of 5 pots within each shadehouse bay.

##### Field evaluation trials

The design for the 2015 field trial was a randomized complete block design where 600 plots were arranged in a 40 row by 15 column array with two replicate blocks comprising rows 1–20 and rows 21–40. Three hundred lines, comprising 276 DYDH lines, two parental lines and 22 other commercial lines were sown in each replicate block. The treatment and blocking structure for the 2015 field trial can be written as

Blocking structure: Block
Treatment structure: Geno

where Block is a factor with 2 levels and Geno is a factor with 300 levels.

The design for the 2016 field trial was a randomized complete block design where 600 plots were arranged in a 50 row by 12 column array with two replicate blocks comprising columns 1–6 and columns 7–12. Two hundred and ninety eight lines, comprising 274 DH lines, two parental lines and 22 other commercial/advanced breeding lines (05-P71-11, ATR-Stingray, AV-Garnet, ATR-Marlin, BC78868-P2, BC78868-P3, BC 78868-P6, BLN3343-CO0402, CB-Telfer, Charlton-093, Crusher-TT, Hyola50, Monty-094, NMT370, Surpass501TT, Tarcoola-27, Tarcoola-43, Tarcoola-22-094, Tarcoola-69-092, Tarcoola-141, Tarcoola-191, and TornadoTT) were sown once in each replicate block. A single commercial line was sown twice in each replicate block. The treatment and blocking structure for the 2016 field trial can be written as

Blocking structure: Block
Treatment structure: Geno

where Block is a factor with 2 levels and Geno is a factor with 299 levels. Each experimental unit (DH/parental/check cv.) was sown in 10 m rows with 50 cm row spacing. Two hundred seed from each line were sown in the blackleg nurseries under lateral move irrigation having mixed TT stubble as described previously (Raman H. et al., 2016).

Expression of QR was assessed at the physiological maturity stage (BBCH 90). Disease severity was assessed as per cent plant survival as well as per cent internal infection for all field and shade house experiments (internal darkening, canker scores) as described previously (Marcroft et al., 2012b; Raman et al., 2012b). Phenological development of DY population (maturity) was assessed in the 2015 field trial based on growth stages using the standard BBCH scale (an ordinal scale between 0 and 9 where 93% of the maturity scores were either: 7, 8, or 9).

### Genetic Mapping

After the assessment of disease severity of the parental lines, ‘Darmor-*bzh*’ and ‘Yudal’ and their DH progenies, fresh leaf samples were obtained in 96 well formatted tubes. Total genomic DNA was isolated following a high throughout DNA protocol, essentially based on chloroform and phenol. A framework map of the DYDH population, based on 2,094 DArTseq markers was constructed as described previously (Raman et al., 2014, 2017). PCR based SCAR (sequence characterized amplified region) marker specific to *Bzh* gene for dwarfness was analyzed using the primer-pairs: SCM07UP1 and LP2 as described in Barret et al. (1998). The quality of marker data was reassessed for its suitability for QTL identification by considering missing data, duplicate markers, markers with excessive segregation distortion, and DH clones that may arise during the microspore culture process. A total of 249 markers with more than 70% missing data, were removed. Two DH lines (DY043 and DY044) which had more than 70% of missing marker scores were also removed from the analysis.

Thirteen DH individuals were removed from the marker data as the proportion of matching genotypes with another marker was more than 99%. The DH individual with the most missing data was the DH individual removed when considering clones. The final set of marker data containing 1842 markers scored as −1, or 1 or NA (missing) over 262 DH lines was used for QTL identification. Missing data for a DH line was imputed using the mean of the 4 nearest neighbors with non-missing data for that DH line using the ASMap (Taylor and Butler, 2016) and pedicure (Butler, 2016) software packages within the R (R Core Team, 2014) computing environment.

### Statistical Analysis of the Phenotypic Data and QTL Mapping

Reliability (broad sense heritability, H^2^) and accuracy (Mrode and Thompson, 2005) were calculated from the extended baseline linear mixed for each trait within trials. The statistical analysis of each trait within a trial used linear mixed model technology and proceeded in six stages: (1) Fit a baseline linear mixed model. (2) Extend the baseline linear mixed model by considering other sources of variation, e.g., spatial variability in field trials. (3) Add markers to the (extended) baseline linear mixed model. (4) Consider each marker separately as a fixed effect in a genome scan model. (5) Based on the genome scan model select a subset of markers upon which backward elimination is performed. (6) A final set of markers is identified and declared as putative QTL. All traits considered, except maturity, were transformed using the logit transformation to better satisfy model assumptions of normality and constant variance.

The baseline statistical model for each trait in each trial can be written, using the symbolic notation described by Wilkinson and Rogers (1973), as trait ∼1 + GnonDH + **GDH** + **BlockingStructure** + **units** where 1 is the overall mean, GnonDH and GDH are a partitioning of the treatment structure term Geno into non doubled haploid lines and doubled haploid lines respectively, i.e., GnonDH indexes the parental, control and commercial lines. The term BlockingStructure refers to factors which reflect trial design. Terms in bold font are considered random terms and associated with each of these terms is a variance parameter (often referred to as a variance component). The preferred method for estimating these parameters is residual (or restricted) maximum likelihood (REML) (Patterson and Thompson, 1971). The term units refer to the residual and its associated variance parameter is the residual variance. Marker effects can be accommodated in the (extended) baseline linear mixed model by partitioning the effects of doubled haploid lines into marker effects plus random genetic variation. In the genome scan model, each marker is fitted as a fixed effect sequentially and for each marker a Wald type conditional *F*-statistic and an associated *p*-value is computed. LOD scores are calculated for each marker using the formula - log10(*p_i_*) where *p_i_* is the *p*-value associated with the *i*-*t*h marker. At the completion of fitting the genome scan model a subset of markers where each marker had a *p*-value less than 0.01, i.e., a LOD score greater than 2, were chosen. Chosen markers that were within 30 cM of another chosen marker were culled to a single representative marker for that region based on LOD score. The chosen subset of markers were simultaneously fitted as fixed effects and backward elimination was conducted by dropping markers fitted as fixed effects one at a time until all remaining markers in the model had a LOD score of approximately 2. This final set of markers is referred to as the putative QTL marker set. The process of performing a genome scan, choosing a subset of markers for backward elimination and identifying a putative QTL marker set is performed a second time with the initial putative QTL marker set fitted as fixed effects in the genome scan and subsequent models. This approach to QTL identification is the approach considered by Nelson et al. (2014). All models were fitted using the statistical software package ASreml (Butler et al., 2009) within the R computing environment.

## Results

### Resistance to *L. maculans* Is Genetically Controlled in DYDH Population

**FIGURE 1:**
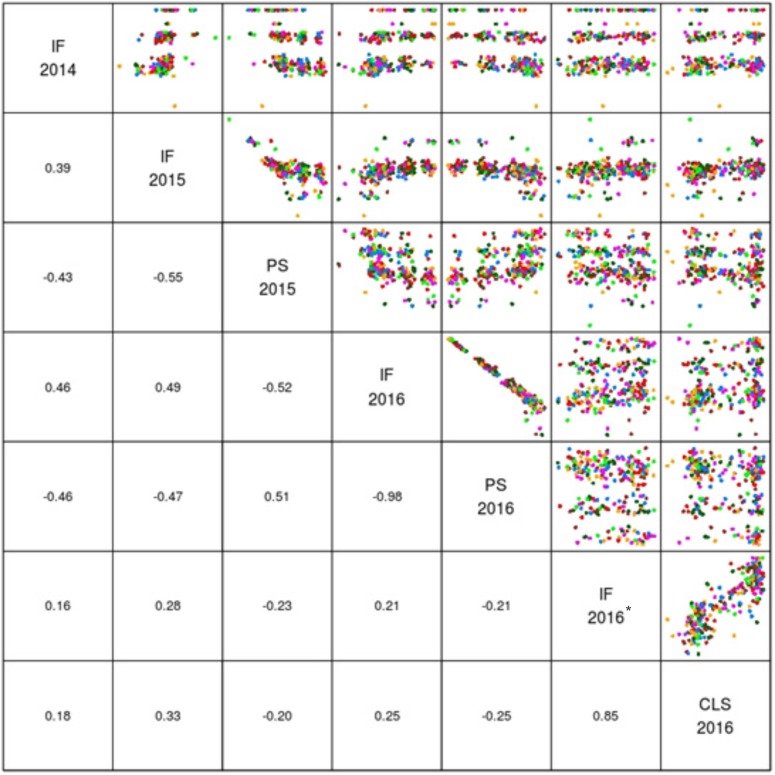
Pair plots showing genetic correlations between different phenotypic scores (EBLUPS) for resistance to *L. maculans* in the doubled haploid population from Darmor-*bzh*/Yudal. CLS, IF, and PS refer to cotyledon lesion score, internal infection and plant survival, respectively. IF 2014 data relate to internal infection scores from ascospore shower test. IF 2016^∗^ relates to internal infection scores from the inoculated plants with PHW1223 isolate.

The mean logit-transformed disease scores for plant survival, cotyledon infection and internal infection (canker scores) were calculated for each phenotypic value. There was a significant genotypic variation for plant survival and internal infection scores among the DH lines. Frequency distributions for plant survival and internal infection showed continuous variation, confirming the quantitative inheritance of resistance to *L. maculans*. The H^2^ varied from 43 to 95%. The accuracy was also generally high for each experiment (Table 1). For example, the values for accuracy of ‘ascospore’ shower test carried-out under shade house were 66, 72 to 84% for field experiments and 88–98% for glasshouse experiments. High values for H^2^ and accuracy suggested that blackleg resistance was controlled by genetic factors, and the phenotypic data could be confidently used for detecting reliable QTL. To determine whether there was any correlation (*r*) between plant survival and internal infection, pair-wise raw correlations were calculated; high negative *r* values were obtained varying from *r* = −0.52 to −0.98 (Figure 1). For internal infection, r values ranged from 0.16 to 0.26 across three experiments (Ascospore shower test, field and glasshouse).

**Table 1:** Estimated reliability (*H*^2^, broad sense heritability) and accuracy from the (extended) baseline linear model for each trait and experiment combination.

Experiment	Trait	#^∗^EBLUP of DH lines (range)	Broad sense heritability (*H*^2^, %)	Accuracy (%)
Ascospore shower test (2014)	^∗^Internal infection score	−5.68 to 3.92	43	66
Field (2015)	^∗^Internal infection score	−6.90 to 7.74	52	72
	^∗^Plant survival (%)	−8.84 to 4.87	56	75
	Maturity	−1.23 to 0.87	58	76
Field (2016)	^∗^Internal infection score	−9.69 to 8.02	71	84
	^∗^Plant survival (%)	−5.46 to 3.62	71	84
Glasshouse (2016): PHW1223 inoculation	Cotyledon lesion	−4.46 to 2.72	95	98
	^∗^Internal infection	−11.36 to 8.80	78	88

*^#^EBLUPS (Empirical Best Linear Unbiased Predictors) were calculated with the ASREML. ^∗^Traits were subjected to logit transformation.*

### QTL Identification: Multiple QTL Control Resistance to *L. maculans*

A total of 31 putative QTL were associated with resistance to *L. maculans* under glasshouse, shade house, and field conditions (Table 2) which were distributed onto chromosomes A02, A04, A06, A07, A08, A09, A10, C01, C02, C04, C07, C08, and C09. No QTL for resistance on chromosomes A01, A03, A05, C03, C05, and C06 was detected in the DYDH population.

**Table 2:** Putative QTL for resistance to *L. maculans* evaluated under different environments.

Phenotyping year	QTL	Phenotyping method	Marker	Chromosome	Genetic map position (cM)	Physical position of markers on Darmor reference genome	Allelic effect	*F.*con	*P* value	LOD score	Genetic variance (*R*^2^) explained (%)	Environment
2015	*Qrlm(ii).wwai-A02a*	Canker	5048589	A02	0.00	245167	−0.99	18.26	0.0000	4.59	5.74	Field
2015	*Qrlm(ii).wwai-A02b*	Canker	3110323	A02	14.98	784222	0.72	16.11	0.0001	4.12	5.10	Field
*2015*	*Qrlm(ps).wwai-A02c*	*Plant survival*	*3103948*	*A02*	*86.41*	*12479164*	*1.54*	*15.08*	*0.0001*	*3.90*	*4.79*	*Field*
**2016**	***Qrim(ps).wwai-A02c***	**Plant survival**	**3194297**	**A02**	**87.22**	**12841291**	**–1.00**	**14.53**	**0.0002**	**3.78**	**4.62**	**Field**
**2014**	***Qrim(ii).wwai-A02c***	**Canker**	**3090338_44:A>T**	**A02**	**87.64**	**18375298**	**1.38**	**19.85**	**0.0000**	**4.93**	**6.21**	**Shade house**
2015	*Qrlm(ps).wwai-A02d*	Plant survival	3088781	A02	90.87	19557827	−0.89	11.21	0.0009	3.04	3.60	Field
2016	*Qrlm(cs).wwai-A02e*	Cotyledon lesion	3140555	A02	126.07	24779399	0.25	9.61	0.0021	2.67	3.10	Glasshouse
2014	*Qrlm(ii).wwai-A04*	Canker	3126287	A04	70.94	19140856	0.84	9.30	0.0025	2.60	3.01	Shade house
2015	*Qrlm(ii).wwai-A06a*	Canker	4338964	A06	24.91	2769171	0.58	9.03	0.0029	2.54	2.92	Field
2014	*Qrlm(ii).wwai-A06b*	Canker	4706344	A06	93.59	20700180	1.06	11.16	0.0009	3.03	3.59	Shade house
2016	*Qrlm(ii).wwai-A06c*	Canker	5120801_7:C>G	A06	105.29	21834553	0.71	8.09	0.0048	2.32	2.62	Glasshouse
2015	*Qrlm(ps).wwai-A06d*	Plant survival	3166196	A06	114.98	23195900	−1.58	33.81	0.0000	7.81	10.13	Field
2016	*Qrlm(ii).wwai-A07a*	Canker	3182957	A07	21.36	10559126	1.14	7.41	0.0069	2.16	2.41	Field
2015	*Qrlm(ii).wwai-A07b*	Canker	3117408_29:G>T	A07	30.00	11417080	0.89	20.19	0.0000	5.00	6.31	Field
**2016**	***Qrim(cs).wwai-A07c***	**Cotyledon lesion**	**3084727_18: A>G**	**A07**	**68.10**	**15955584**	**1.87**	**366.90**	**0.0000**	**16.00**	**55.02**	**Glasshouse**
**2016**	***Qrim(ii).wwai-A07c***	**Canker**	**5048102_5:A>T**	**A07**	**68.50**	**16024054**	**5.33**	**459.30**	**0.0000**	**16.00**	**60.49**	**Glasshouse**
2015	*Qrlm(ii). wwai-A07d*	Canker	4118297	A07	112.96	22499718	0.46	6.57	0.0109	1.96	2.14	Field
2015	*Qrlm(ii).wwai-A08*	Canker	3113258_62:A>G	A08	50.29	14937354	0.51	7.85	0.0054	2.27	2.55	Field
*2014*	*Qrlm(ii).wwai-A09a*	*Canker*	*3104830_27:A>G*	*A09*	*53.76*	*8115728*	*0.89*	*8.23*	*0.0044*	*2.35*	*2.67*	*Shade house*
*2015*	*Qrlm(ps).wwai-A09a*	*Plant survival*	*3128922*	*A09*	*54.97*	*8250906*	*–1.06*	*13.60*	*0.0003*	*3.57*	*4.34*	*Field*
**2016**	***Qrim(ii).wwai-A09b***	**Canker**	**3128015**	**A09**	**71.09**	**21101959**	**1.51**	**12.10**	**0.0006**	**3.24**	**3.88**	**Field**
**2016**	***Qrim(ps).wwai-A09b***	**Plant survival**	**3128015**	**A09**	**71.09**	**21101959**	**–1.10**	**15.71**	**0.0001**	**4.03**	**4.98**	**Field**
**2016**	***Qrim(ii).wwai-A10a***	**Canker**	**3101711**	**A10**	**70.58**	**158471178**	**1.17**	**7.75**	**0.0057**	**2.24**	**2.52**	**Field**
**2016**	***Qrim(ps).wwai-A10a***	**Plant survival**	**3101711**	**A10**	**70.58**	**15847118**	**–0.91**	**11.53**	**0.0008**	**3.11**	**3.70**	**Field**
2015	*Qrlm(ii).wwai-A10b*	Canker	4114338	A10	75.85	16682012	0.49	7.01	0.0085	2.07	2.28	Field
2016	*Qrlm(ii).wwai-C01a*	Canker	3167015	C01	49.56	13113078	−0.76	9.29	0.0025	2.60	3.00	Glasshouse
2015	*Qrlm(ps).wwai-C01b*	Plant survival	4168991	C01	61.32	36835698	0.97	11.46	0.0008	3.09	3.68	Field
**2016**	***Qrim(ii).wwai-C01c***	**Canker**	**5121627**	**C01**	**100.17**	**37068377**	**–1.10**	**7.39**	**0.0069**	**2.16**	**2.40**	**Field**
**2016**	***Qrim(ps).wwai-C01c***	**Plant survival**	**3111753_53:C>A**	**C01**	**101.80**	**37068377**	**0.76**	**9.08**	**0.0028**	**2.55**	**2.94**	**Field**
2016	*Qrlm(ii).wwai-C02a*	Canker	3141726	C02	84.21	19785381	−1.17	21.11	0.0000	5.19	6.57	Glasshouse
2016	*Qrlm(cs).wwai-C02b*	Cotyledon lesion	4107313	C02	135.68	42431361	0.22	5.61	0.0185	1.73	1.84	Glasshouse
2014	*Qrlm(ii).wwai-C04*	Canker	3096611_34:T>G	C04	84.30	38312161.5	1.02	12.63	0.0004	3.36	4.04	Shade house
2014	*Qrlm(ii).wwai-C07*	Canker	5050300	C07	92.30	43262249.5	0.90	9.89	0.0018	2.74	3.19	Shade house
2015	*Qrlm(ps).wwai-C08*	Plant survival	3183817	C08	32.57	20766069	−0.80	10.31	0.0015	2.83	3.32	Field
2016	*Qrlm(ii).wwai-C09a*	Canker	3141407	C09	12.22	1296279.5	−0.90	11.78	0.0007	3.17	3.78	Glasshouse
*2016*	*Qrlm(cs).wwai-C09b*	*Cotyledon lesion*	*3144245_23:T>C*	*C09*	*55.06*	*3600000*	*0.27*	*9.42*	*0.0023*	*2.63*	*3.05*	*Glasshouse*
*2016*	*Qrlm(ii).wwai-C09c*	*Canker*	*3092936*	*C09*	*57.14*	*34154278*	*–1.00*	*6.08*	*0.0142*	*1.85*	*1.99*	*Field*
**2016**	***Qrim(ii).wwai-C09d***	**Canker**	**5120888**	**C09**	**93.42**	**45405183**	**1.17**	**12.16**	**0.0006**	**3.25**	**3.90**	**Field**
**2016**	***Qrim(ps).wwai-C09d***	**Plant survival**	**5120888**	**C09**	**93.42**	**45405183**	**–0.75**	**12.11**	**0.0006**	**3.24**	**3.88**	**Field**
**2014**	***Qrim(ii).wwai-***	**Canker**	**3079341**	**C09**	**104.51**	**46622430.2**	**0.81**	**9.04**	**0.0029**	**2.54**	**2.93**	**Shade**
	***COe***											**house**
**2015**	***Qrim(ii).wwai-COe***	**Canker**	**3079341**	**C09**	**104.51**	**46622430.2**	**0.85**	**28.00**	**0.0000**	**6.63**	**8.54**	**Field**
**2015**	***Qrim(ps).wwai-C09e***	**Plant survival**	**3079341**	**C09**	**104.51**	**46622430.2**	**–1.05**	**23.02**	**0.0000**	**5.60**	**7.13**	**Field**

*Wald Type conditional F-statistics (F-con), P-value and LOD score, estimated genetic variation (%) by each QTL is presented. Resistance to blackleg was measured as per cent internal canker infection (ii, canker) as well plant survival (ps). Consistent QTL that appeared across experiments are highlighted in Bold letters and loci that mapped within 2cM are highlighted in Italics letters. Cotyledon lesion scores (cs) represent to single spore isolate, PHW1223 mediated infection. QTL nomenclature was followed as described previously in Raman et al. (2012b). Allelic effects in + and -ve directions represent to Darmor and Yudal resistance alleles, respectively.*

#### Glasshouse Experiments

**FIGURE 2:**
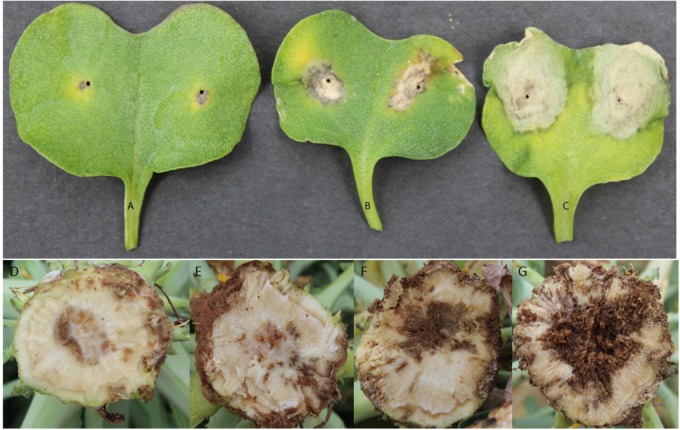
**(A–C)** Cotyledons of doubled haploid lines showing lesions upon inoculation with *Leptosphaeria maculans* isolate PHW1223. Symptoms were assessed after 20 days of infection. **(A,B)** resistant DH lines and **(C)** susceptible line. **(D–G)** Varying levels of internal infection among doubled haploid lines of Darmor-*bzh*/Yudal population assessed at the physiological maturity stage.

**FIGURE 3:**
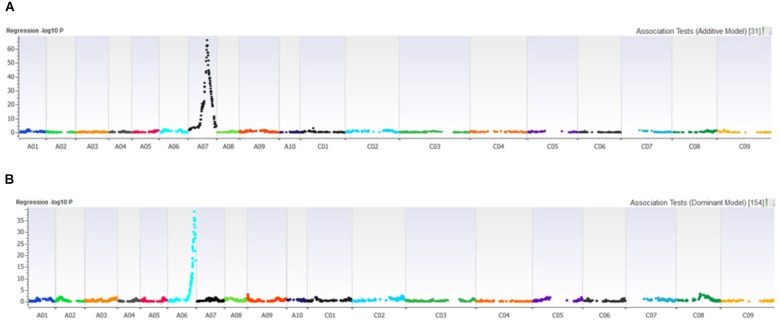
Manhattan plots showing association for **(A)** resistance to PHW1223 isolate of *L. maculans* assessed at the cotyledon stage after 20 days of inoculation, and **(B)** for dwarfness (*bzh*) in the Darmor-*bzh*/Yudal DH population.

**FIGURE 4:**
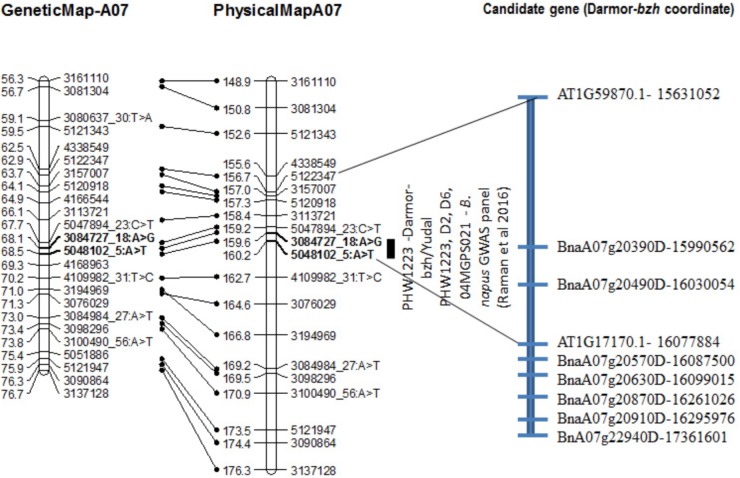
Genetic and physical localization of *Rlm9* locus for resistance to *L. maculans* in a doubled haploid population from Darmor-*bzh*/Yudal. Physical map positions are based on the reference genome assembly of Darmor*-bzh* (Chalhoub et al., 2014). Markers that showed highly significant association with resistance to PHW1223 isolate (*AvrLm9*) at both seedling and adult plants stages are in bold letters. For clarity, both partial genetic (in cM) and physical maps of chromosome A07 are shown herein; physical locations are depicted as 1/100,000th from the original coordinates. For details, see Supplementary Table 3.

Distribution of scores obtained for 259 DH lines of DY population using PHW1223 isolate of *L. maculans* following randomized complete block design are shown in Supplementary Figure 1. We identified three QTL for resistance to *L. maculans* evaluated at cotyledonary stage, on chromosomes A02, A07 and C09 (LOD ≥ 2) accounting for 3.05–55% of genotypic variation (Table 2). In addition, a suggestive QTL with LOD score of 1.7 was also detected on C02. Of these, QTL; *Qrlm(cs).wwai-A07c* on chromosome A07 detected with DArTseq-SNP marker; 3084727_18:A>G explained the majority (87.33%) of the total genotypic variance (Supplementary Table 1). ‘Darmor-*bzh*’ contributed alleles for resistance at all three QTL regions. Since the QTL on A07 accounted for the largest *R*^2^, we binned the quantitative phenotypic data into two categories: resistant and susceptible based on the size of lesions on cotyledons (Figure 2A) as described previously (Raman et al., 2012a,b). 122 DH lines were resistant, and 134 lines were susceptible to isolate PHW1223 of *L. maculans*, suggesting that segregation of resistance and susceptibility conforms to monogenic ratio (χ^2^ = 0.56; *P* = 0.45). Using this binned data, a major gene for resistance could be mapped on chromosome A07 (Figure 3A). We deduced that *Rlm9* gene control resistance in the DY population on the basis of *avr* gene profile of *L. maculans* isolate (*AvrLm5, AvrLm6, AvrLm8, AvrLm9, LepR3*), and is consistent with the previous study (Delourme et al., 2004). To verify whether disease expression for resistance is not as a result of experiment ‘escape,’ all resistant DH lines were inoculated with the D13 isolate carrying *avrLm9*. Resistant DH lines to PHW1223 showed susceptible reaction to this isolate. This confirmed that all resistant phenotypes were correct. When the inoculated seedlings of DH lines with isolate PHW1223 were raised to physiological maturity under glasshouse conditions at Wagga Wagga and subsequently evaluated for the extent of internal infection, five QTL were detected on chromosomes A06, A07, C01, C02 and C09, accounting for 2.62–60.49% of genetic variance (Table 2). At these genomic regions, allelic effects on A06 and A07 were contributed by ‘Darmor-*bzh*,’ while ‘Yudal’ contributed alleles for resistance at C01, C02, and C09. Among different genomic regions detected, the *Qrlm(cs).wwai-A07c* on A07 explained the largest *R*^2^ (60.49%) and corresponded to the *Rlm9* location (Figure 4), suggesting that *Rlm9* mediated resistance was expressed at the adult plant stage under glasshouse conditions.

#### Ascospore Shower Test

Seven QTL were detected for internal infection under shade house conditions which were located on chromosomes A02, A04, A06, A09, C04, C07, and C09 (Table 2). These QTL accounted for 25.6% of total genotypic variation (*R*^2^) for internal infection; each QTL explained from 2.67% (on A09) to 6.21% (on A02) of the variation (Supplementary Table 1). The resistant parent, ‘Darmor-*bzh*’ contributed resistance alleles at all seven QTL regions. Of check cultivars, only ‘ThumperTT’ had less internal infection (0–10%) compared to other check cultivars; ATR-Marlin, ATR-Stingray, CB-Telfer, Crusher, and Hyola444.

#### Field Experiments

DH lines were evaluated for plant survival, internal infection and plant maturity (in 2015) across 2 years and showed continuous variation for resistance (Figure 1 and Supplementary Figure 2). Results on putative QTL and their allelic effects are presented in Table 2 and Supplementary Table 1. For plant survival, 11 QTL were detected on chromosomes A02, A06, A09, A10, C01, C08, and C09, explaining 2.94–10.13% of genotypic variance. The *Qrlm(ps).wwai-A06d* delimited with marker 3166196 on A06 accounted for the largest proportion (10.13%) of the variation. Across both experiments, at least one QTL flanked by DArTseq markers; 3194297–3088781 on chromosome A02 was detected repeatedly. Both parental lines, ‘Darmor-*bzh*’ and ‘Yudal’ contributed favorable alleles for resistance to *L. maculans*; ‘Darmor-*bzh*’ contributed alleles for resistance at A02, A06, A09, A10, C08, and C09, while ‘Yudal’ contributed resistance allele at QTL on chromosome C01 in 2015.

For internal infection, 14 QTL were detected on chromosomes A02, A06, A07, A08, A09, A10, C01, and C09 (Table 2). These QTL explained 1.99–8.54% of the genotypic variance. QTL delimited with 3079341 marker (LOD score = 6.63) accounted for the largest variation. In 2015, only maternal parent, ‘Darmor-*bzh*’ contributed favorable alleles for resistance whereas both parents of DH population contributed resistance alleles in 2016. Allelic effects contributed by ‘Yudal’ were only detected at two QTL on chromosomes C01 and C09.

### Consistent QTL Across Phenotypic Environments

**FIGURE 5:**
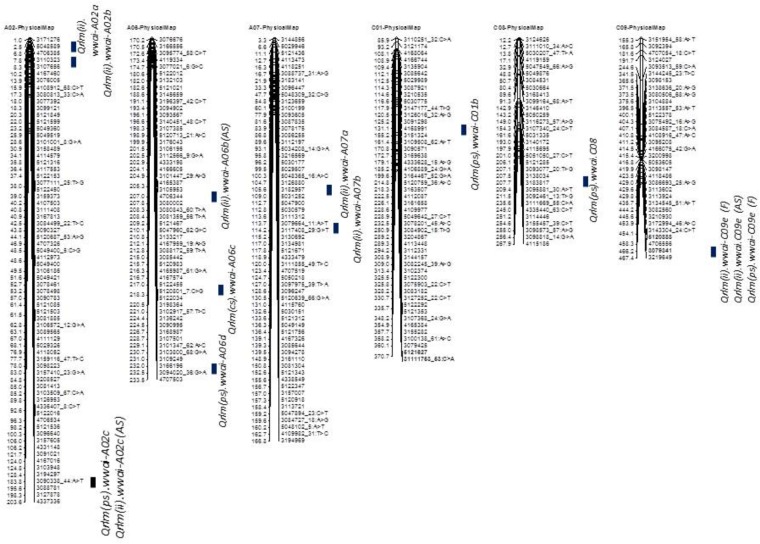
Localization of ‘stable’ QR loci for resistance to *L. maculans* on the physical map of *B. napus* cv. Darmor-*bzh.* QTL for QR were identified in a doubled haploid population derived from Darmor-*bzh*/Yudal. Resistance was tested as (i) internal infection under shadehouse conditions with ascospore shower test [2014-IF (AS)], (ii) internal infection under field conditions in 2015 and 2016 [2015/2016-IF (F)], and (iii) plant survival under field conditions in 2015 and 2016 [2015/2016-PS (F)]. Race-specific resistance was evaluated with a single spore isolate, PHW1223 of *L. maculans*. Only stable QTL that were detected for QR (this study and Kumar et al., 2018) are shown herein. Physical locations are depicted as 1/100,000th from the original coordinates.

Taken together, of the 31 genomic regions for resistance to *L. maculans* identified, nine were consistently detected across different experiments on chromosomes A02, A07, A09, A10, C01, and C09; four QTL were common between internal infection and plant survival (on A02, A10, C01, and C09), three were common between two field experiments (on A02, A10, and C09) and two QTL were common between ascospore shower and field test (on chromosomes A02, and A09). Only one QTL on chromosome C09 was detected for resistance assessed by three phenotypic methods: plant survival, internal infection under field conditions and ‘ascospore’ shower test (Figure 5 and Table 2). None of the QTL significantly associated with internal infection and plant survival, assessed either using the ascospore shower or field tests colocalized with *Rlm9*.

### Localization of Stable QR Loci to *L. maculans* Across Environments

To identify QR loci of global relevance, we compared QTL that were detected in the DYDH population for resistance evaluated under different phenotypic environments in France, United Kingdom, and Australia (Kumar et al., 2018), based on their physical map positions of QTL regions on the reference ‘Darmor-*bzh*’ genome assembly (Chalhoub et al., 2014). Physical coordinates that were mapped within ∼200 Kb were considered as consistent/stable; i.e., the same QTL across environments. A total of 8 genomic regions that were located on chromosomes A02, A06, A07, C01, C08, and C09, were repeatedly detected in different environments: Australia, France and/or United Kingdom (Supplementary Table 2). These QTL were unevenly distributed across the *B. napus* genome, two QTL on chromosome A02 and A06, and one each on chromosomes A07, C01, C02 and C09. Resistant alleles at QTL mapped on chromosome C01 in both Darmor-*bzh*/Yudal and Darmor-*bzh*/Bristol DH populations are contributed by the susceptible parent (Yudal or Bristol, Kumar et al., 2018), were also detected in our study (Supplementary Table 2).

### Mapping of QTL for Plant Maturity

In order to establish whether there was any link between plant maturity and resistance to *L. maculans*, we phenotyped the DYDH population for plant maturity in the 2015 trial using BBCH scoring system. Our results showed that DH lines display a continuous variation in plant maturity (Supplementary Figure 2), implying its quantitative inheritance. Seven putative QTL were detected for plant maturity on chromosomes A02, A06, C01, C02, C06, and C09, accounting for the total of 57.60% of *R*^2^ (Supplementary Table 1). The QTL on C09 (LOD = 12.7) explained the largest proportion (16.49%, >30%) of the total variation^,^ followed by QTL on C02. Both the parents of DH population accounted for genotypic variation in plant maturity; ‘Darmor-*bzh*’ alleles accounted for late maturity at QTL on A02, A06, C02, and C09 chromosomes, while early maturity alleles at QTL on A06, C01, and C06 were contributed by parent, ‘Yudal.’ Three significant QTL associated with plant maturity were located near the QR loci on A02, A06, and C09, suggesting that these genomic regions may have a pleiotropic effect on QTL for QR (Supplementary Table 1).

### Mapping of *Bzh* Gene for Dwarfness

In order to establish whether there was any relationship between plant height (dwarfness due to *bzh*) and QR loci to *L. maculans*, we conducted molecular screening for *bzh* gene using gene specific PCR based markers (Foisset et al., 1995). Of 148 DH lines tested, 79 had 439 bp allele specific for dwarfness (Darmor-*bzh* type) and 69 lines had 528 bp allele for non-*bzh* (Yudal type) (Supplementary Figure 3). Chi squared test revealed that the allelic segregation was not significantly different from 1:1 ratio (χ^2^ = 0.676; *P* = 0.4111). This is consistent with ‘Darmor-*bzh*’ and DH progeny from the Darmor-*bzh*/Yudal having *bzh* gene. Linkage analysis reconfirmed that *bzh* marker was indeed mapped on A06, consistent with chromosomal location of the *bzh* gene (Foisset et al., 1995). PCR marker showed tight linkage with 3103800_68:G > A (LOD = 38.97) and 3101347_62:A > C (LOD = 36.19) markers which were mapped on chromosome A06 (Figure 3B). Alignment between DArTseq marker sequences linked with *bzh*, and the genome sequences of Darmor-*bzh* showed that *bzh* locus is inferred to reside within a region of 22.9 Mb on A06. One of the QTL for plant maturity was mapped close (34.5 kb) to the *bzh* genomic region. In addition, one QTL associated with plant survival (in the 2015 trial) was also located within 121 Kb from *bzh* gene. Location of QTL for plant dwarfness, physiological plant maturity and QR at A06 region delimited with 22.8–23.1 Mb coordinates on the Darmor-*bzh* genome further hints toward the possible role of plant developmental genes in blackleg resistance in canola (Raman H. et al., 2016).

### Physical Mapping and Identification of Candidate Genes Underlying QR and *Rlm9*

Release of the reference genome assembly of cv. Darmor*-bzh* (Chalhoub et al., 2014) enabled us to identify location of trait marker associations on the physical map of canola, representing both A and C subgenomes (Supplementary Table 3). The current annotation of the ‘Darmor-*bzh*’ reference genome assembly (v4.0) predicted at least five *B. napus R* genes, which were mapped within 100 Kb from ten significant marker-trait associations that were detected in the DYDH population, evaluated using ascospore shower test (2014), internal infection and disease severity, and plant survival (2015) (Supplementary Table 3). Predicted *R* genes in the vicinity of QR loci included BnaA02g015160D (A02), BnaA020120D (A02), BnaA07g04380D (A07), BnaA09g14320D (A09), BnaC09g02130D (C09), and BnaC09g06020D (C09), BnaC09g44520D (C09) having TIR - NBS – LRR (Chalhoub et al., 2014; Alamery et al., 2018). Two marker associations detected in the 2016 glasshouse (PHW1223 isolate) and 2015 field experiment (plant survival) were mapped within 6 Kb from resistance genes (Supplementary Table 3). DArTseq marker 3141726 (A09) was localized 2.94 Kb from a homolog of AT1G54470.2 which is involved in resistance to several isolates of *Peronospora parasitica* causing downy mildew resistance, while marker 3128922 (C02) was localized within 5.8 Kb from *B. napus* resistance gene BnaA09g14420D, having TIR - NBS - LRR motif (Alamery et al., 2018). In addition, LRR and NB-ARC domains-containing disease resistance protein gene (AT1G61180.2) was located ∼102 Kb from the QTL for internal infection (ascospore shower test) on chromosome C04.

The *Rlm9* locus was localized at the coordinates 15,955,583–16,024,053 bp (Figure 4) of the ‘Darmor-*bzh*’ genome assembly on chromosome A07 and mapped in the vicinity of BnaA07g20390D and BnaA07g20490D. Several genes implicated in disease resistance such as BnaA07g20630D (AT1G78290.2, Protein kinase superfamily protein), BnaA07g20870D (AT1G77700.1, Pathogenesis-related thaumatin superfamily protein) and BnaA07g20910D (AT1G21870.1, golgi nucleotide sugar transporter 5) and BnA07g22940D were located near *Rlm9*.

## Discussion

### Multiple Genes Control QR in the DY Population

Our results first confirm that ‘Darmor’ QR is effective in Australian conditions, despite the composition of *L. maculans* population is different from the one in Europe (Light et al., 2011; Rouxel and Balesdent, 2017), thus confirming the broad-spectrum of this resistance. Continuous segregation for resistance to *L. maculans* was observed among DH lines assessed on the basis of disease severity (plant survival and internal infection). High *H*^2^ values for field trials are comparable with those recently reported in ‘Darmor-*bzh*/Yudal’ population (Kumar et al., 2018) for QR, which implied that observed variation in resistance is highly heritable in different conditions such as the blackleg nurseries under field conditions across years as well as in ascospore shower test. Therefore, genetic gain could be made for QR to blackleg in canola improvement programs. There were lower *H*^2^ and accuracy values for ‘ascospore shower’ test as compared to field experiments (Table 1). This could be due to the G × E interactions and inoculation method. It is mentioned that we inoculated DH lines with ascospore shower test initially, and once the symptoms appeared on susceptible check cultivars and on some of the DY lines, plants were then vernalised under cold conditions (4°C for 11–12 weeks). Such conditions may have also partly contributed to G × E interactions. We identified a total of 27 QTL associated with quantitative resistance to *L. maculans* under shade house and field conditions, which were distributed on 12 chromosomes, except on A01, A03, A05, C03, C05, and C06 (Table 2). These observations confirmed that quantitative resistance to blackleg is due to multiple genes distributed across genome.

### Stable QTL for QR Across Environments

We detected 15 significant QTL (LOD score ≥ 3.0) for QR across different experiments. At least four genomic regions on A02, A09, A10, and C09 were repeatedly detected for resistance evaluated as internal infection and plant survival under field/ascospore test, suggesting that some resistance mechanisms are consistently expressed across environments. However, we also found that detection of QTL and their magnitudes were highly dependent upon phenotyping environment. For example, of 13 QTL detected for internal infection, only one appeared repeatedly across 2 years. This could be attributed to several environmental factors which may have influenced the expression and intensity of *L. maculans* under field conditions. In addition, a range of *r*^2^ values, 36.6–66.1% were accounted for by QTL (Kumar et al., 2018), whereas such values ranged from 20.1 to 37.9% in our study. Such discrepancy could also be due to growing environment and G × E interactions.

In an independent study, Kumar et al. (2018) reported 16 QTL for resistance in this population on 15 chromosomes but not on A10, C02, C03, and C05. In both described studies, there was no evidence of QR loci on chromosome C03 and C05. In order to identify loci for QR relevant internationally, we compared the physical location of QTL (significant marker associations/confidence marker intervals) that were detected in this and previous studies. A total of 8 genomic regions that were located on chromosomes A02, A06, A07, C01, C02, and C09 were repeatedly detected in different environments: Australia, France, and United Kingdom. On chromosome A10, we identified two genomic regions, one within 1.42 Mb from BnaA10g24700D gene for resistance to *LepR3/Rlm2* complex, and second near the BnaA10g25550D (Darmor coordinates 16,682,012 bp). Both QTL were not detected in Darmor-*bzh*/Yudal population in France (Kumar et al., 2018) or United Kingdom (Huang et al., 2016) conditions. Discrepancy in detecting QTL could be attributed to the method used for QTL identification in different studies as well as phenotypic environments. However, many of the putative QTL identified in this study are in similar positions to those reported earlier (Huang et al., 2016; Kumar et al., 2018). An example is the stable QTL reported on A02. Another putative QTL identified in this study on C01 for a number of trials and trait combinations may also be of interest to canola breeding programs as it appears in one of the trials (RREs08) reported by Huang et al. (2016). We found that the majority of favorable alleles for resistance were contributed by ‘Darmor-*bzh*’ at QR loci, while only two QTL on chromosomes C01 and C09 were detected in ‘Yudal.’ Our results are consistent with Kumar et al. (2018) who reported resistance alleles from ‘Yudal’ on A01, A03, and C01. Though in our study, no QTL for quantitative resistance was identified on A01 and A03 in the Darmor-*bzh*/Yudal DH population, QTL for resistance to *L. maculans* on chromosome A01 have been reported previously in the Skipton/Ag-Spectrum, Ag-Castle/Topas, Damor-*bzh*/Yudal and diversity panels of *B. napus* (Raman et al., 2012b; Raman H. et al., 2016; Larkan et al., 2016a; Kumar et al., 2018) Further research is required to fine map these loci to understand their structure, function and evolution.

It was interesting to note that despite non-vernalisation of DH lines derived from winter (‘Darmor-*bzh*’) and spring (‘Yudal’) cross and limited plant growth in 56 well tray having 35 × 35 × 35 mm dimension (length, width, and height) cells, several QTL for internal infection were detected at the vegetative stage, however, none of them was consistently detected under field conditions (after 28 weeks of sowing). It remains to be established how far G × E interaction plays a role in differential expression for QR under field and glasshouse conditions.

### QR Locations vs. QTL Associated With Plant Maturity and Plant Height

A major QTL for QR explaining 10.13% of total genetic variance on A06 was located near the genomic region that showed significant association with maturity (∼322 Kb) and dwarfness (∼121 Kb). The second ‘major’ QTL for QR was mapped on chromosome C09 and was also located near the genomic position of plant maturity QTL, accounting for 16.5% of total variance. These findings suggested that QR may be partly conditioned by plant developmental genes as suggested previously (Raman H. et al., 2016). In previous studies, QTL for QR have been mapped near the *bzh* locus for dwarfness on chromosome A06 under French (INRA 96 trial) and United Kingdom field (RRes09) conditions (Pilet et al., 1998; Huang et al., 2016).

### QR Genes May Correspond to ‘Weak’ Expression of *R* Genes

None of *L. maculans R* genes either cloned (*LepR3/Rlm2*) or mapped on the reference Darmor-*bzh* genome assembly (Larkan et al., 2013, 2015, 2016b; Raman H. et al., 2016), were located within 200 Kb of QR loci. However, several QR loci with moderate to small allelic effects were localized in the vicinity of possible qualitative *R* genes. Our results showed that at least 8 QR loci identified in ascospore shower test and field conditions for plant survival and internal infection do map within 1.9–167.2 Kb (Supplementary Table 3) from the genomic regions detected from cotyledon tests with single spore isolates (IBCN17, IBCN18, IBCN75, and PHW1223) on chromosomes A06 and C09 in our previous study (Raman H. et al., 2016). C09 QTL (coordinate 46,622,430) for QR detected with ascospore shower test, internal infection and plant survival was mapped 24.1 Kb apart from the genomic region identified with cotyledon tests using IBCN17 and PHW1223 isolates in a *B. napus* GWAS panel. Another C09 QTL (coordinate 45405183) for QR detected in field for plant survival and internal infection was mapped within 1.9 Kb from the genomic region identified with IBCN75 isolate in the same GWAS panel. A suite of five SNP markers (4113953|F| 0-39:C > T, 3098403|F| 0-41:C > A, 100061301|F| 0-62:A > G, 3099981|F| 0-28:G > T, 3168084|F| 0-9:G > A) associated with internal infection on A06 were detected within 187.1 Kb from the genomic region detected with ascospore shower test (ATR-Cobbler) as well as with cotyledon test using isolate, PHW1223 in the GWAS panel (Raman H. et al., 2016). Colocalization of genomic regions detected with ascospore shower, plant survival, internal infection and single spore isolates suggests that QR resistance to *L. maculans* may be partly conditioned by *R* genes which may have residual effect, or by other genes linked to *R* genes (Chantret et al., 1999; Delourme et al., 2004). This hypothesis is only partly supported by our glasshouse experiment, where we challenged the DY lines with a single spore isolate PHW1223 and assessed resistance at the cotyledon and adult plants stages. We detected three QTL for resistance to *L. maculans* on chromosome A02, A07 and C09 using quantitative lesion scores on cotyledon. Only one QTL detected on C09 genomic regions with single spore isolate PHW1223 showed significant association with resistance to *L. maculans* under field conditions but resistant allele was not contributed by the same parent. Thus whether this C09 QTL region is also involved in QR remains unclear.

A quantitative trait locus from the glasshouse experiment mapped on A07 corresponds to *Rlm9*. Physical location of *Rlm9* gene in the ‘DYDH’ population is consistent with the one in BC_1_F_1_ derived population between *Rlm9*-introgression line/Topas (Larkan et al., 2016b). The efficacy of *Rlm9* was assessed in the field as well as under glasshouse conditions. *Rlm9* did not provide any resistance to *L. maculans* in the field, suggesting that virulent races (*avrLm9*) attacking *Rlm9* were present in our Australian field conditions (Light et al., 2011). However, when the efficacy of *Rlm9* (assessed at cotyledon stage) was tested at the adult plant stage under glasshouse conditions, *Rlm9* conferred complete resistance. These results imply that *Rlm9* gene indeed confers resistance at the adult plant stage provided there is corresponding *AvrLm9* gene present in *L. maculans*. Our results are consistent with the previous genetic analyses studies which showed that the *R* gene mediated resistance expressed at the cotyledon stage is also effective at the adult plant stages in *B. napus* populations, grown under controlled/field environment conditions in the presence of specific corresponding *Avr* gene (Delourme et al., 2004; Raman et al., 2012a,b). However, we found that other significant QTL (e.g., C02 and C09) account for QR in the DY population, besides the *Rlm9* gene on chromosome A07. This is not fully consistent with single gene for gene interaction. It is possible that other uncharacterized *Avr* genes (and/or interaction) may exist in the differential set of Australian *L. maculans* isolates which are not identified yet and which are ‘poorly’ recognized by the plant. In a previous study, Delourme et al. (2004) identified two QTL for resistance to *L. maculans* isolate, IBCN56 (*AvrLm1*, *AvrLm2*, *AvrLm3*, *AvrLm5*, *AvrLm6*, *AvrLm9*) in ‘Darmor-*bzh*/Yudal’ population: a major QTL was mapped on LG10 (A07) corresponding to *Rlm9* and a minor QTL on LG16 (A10, *R*^2^ = 9%) at a position corresponding to *Rlm2/LepR3* (Delourme et al., 2004). The later region also showed association with resistance at the adult plant stage (Pilet et al., 2001). In this study, we did not find any evidence for QTL associated with resistance at the seedling (cotyledon) stage on A10 using PHW1223 isolate (*AvrLm5, AvrLm6, AvrLm8, AvrLm9, LepR3)*. However, we identified QTL for internal infection and plant survival on A10 which was mapped 1.4 Mb part from *LepR3/Rlm2* genes, as in Pilet et al. (2001).

Expressions of weak gene/partial resistance could also be due to the growing environment. For example, we reported a significant QTL on chromosome A01 for blackleg resistance in the Skipton/Ag-Spectrum DH population (Raman et al., 2012b), which was later on validated in the Skipton/Ag-Spectrum//Skipton as well as in *B. napus* diversity panels as having a major allelic effect (Raman et al., 2012b; Larkan et al., 2016a; Raman H. et al., 2016). It remains to be established whether some *R* genes for *L. maculans* resistance which do not express or partially express at the seedling stage, become more effective at the adult plant stage, as shown in Cf-9B/L. system (Panter et al., 2002).

### Putative Candidate Genes for Resistance Loci

Physical localization of *R* genes from *A. thaliana*, and *B. napus* in the vicinity of significant trait-marker association suggested that these genomic regions may be associated with defense related genes. For examples, *B. napus R* genes such as BnaA02g25160D with CC – NBS – LRR domain, BnaA02g36900D with TIR domain, BnaA09g14320D, BnaC09g06020D and BnaC09g44520D with TIR – NBS – LRR domains and *A. thaliana R* genes such as AT2G05940.1 (*RPM1*-INDUCED PROTEIN KINASE) which encodes a receptor-like cytoplasmic kinase that phosphorylates the host target *RIN4*, leading to the activation of a plant innate immune receptor *RPM1*, were detected within 200Kb from significant SNP associations. AT1G56510.1 (BnaC09g06020D) is reported to confer resistance to races of *Albugo candida*. The TIR, NBS, LRR, protein kinase proteins are well known to confer disease resistance in plants (Césari et al., 2014).

Recently, several transcripts involved in *L. maculans* infection in resistant and susceptible lines were identified in *B. napus* lines upon infection with *L. maculans*, at the cotyledonary stage (Becker et al., 2017). We compared the physical position of those genes with the genomic regions associated with resistance in DY population. We found two genes, BnaA07g32130D and BnaC01g36910D within 200 Kb from the transcripts which are shown to be accumulated upon infection with *L. maculans* (Becker et al., 2017). The BnaA07g32130D (PDF1.2) gene model relates to the *A. thaliana* gene (AT5G44420.1) and belongs to the plant defensin (PDF) family and encodes an ethylene- and jasmonate-responsive plant defensin, while the BnaC01g36910D was mapped within 100 Kb from a significant QTL for field canker and survival on C01.

### Strategies to Incorporate Into Breeding Programs

Various breeding programs prefer to introgress qualitative and quantitative resistance genes with major allelic effects to *L. maculans*. In this study, we found eight significant QTL regions for QR on A02, A07, A09, A10, C01, and C09 which were detected across diverse growing environments: Australia, France, and United Kingdom. Almost all QTL had small allelic effects and accounted for less than 10% of the genotypic variance individually. Some of the allelic effects have been validated in DH populations derived from ‘Darmor-*bzh*/Bristol’ and ‘Darmor/Samourai’ and diversity panels. Therefore, these validated and stable loci can be introgressed for enhancing QR in elite canola germplasm via molecular marker assays such as KASP and sequence capture probes. Given that QR is difficult to select and confounded with environmental factors, genomic selection could be performed while also targeting loci associated with high grain yield, flowering time and tolerance to water and nutrient stress in the canola improvement programs.

## Author Contributions

HR and RR planned the experiments and wrote the manuscript. RD and SD edited the manuscript. DB, SM, and PS carried-out ascospore shower test at Horsham. HR, RR, and YQ carried out single spore and field experiments. HR and RR scored all field, glasshouse, and CE trials. SD and HR performed genetic/QTL analyses. BM screened the marker for *bzh* in the DY population. All authors read and commented on manuscript.

## Conflict of Interest Statement

The authors declare that the research was conducted in the absence of any commercial or financial relationships that could be construed as a potential conflict of interest.
