# Supplementary material for: Stable Quantitative Resistance Loci to Blackleg Disease in Canola (Brassica napus L.) Over Continents
Source: Front Plant Sci. 2018 Nov 23;9:1622. doi: 10.3389/fpls.2018.01622 (PMC6265502; doi:10.3389/fpls.2018.01622)

Supplementary Figure 2: Distribution of maturity scores (EBLUPS) in the Darmor-*bzh*/Yudal DH population evaluated under disease nursery (field conditions) in 2015 at Wagga Wagga. DH lines were grown on stubble from open pollinated mixed TT varieties

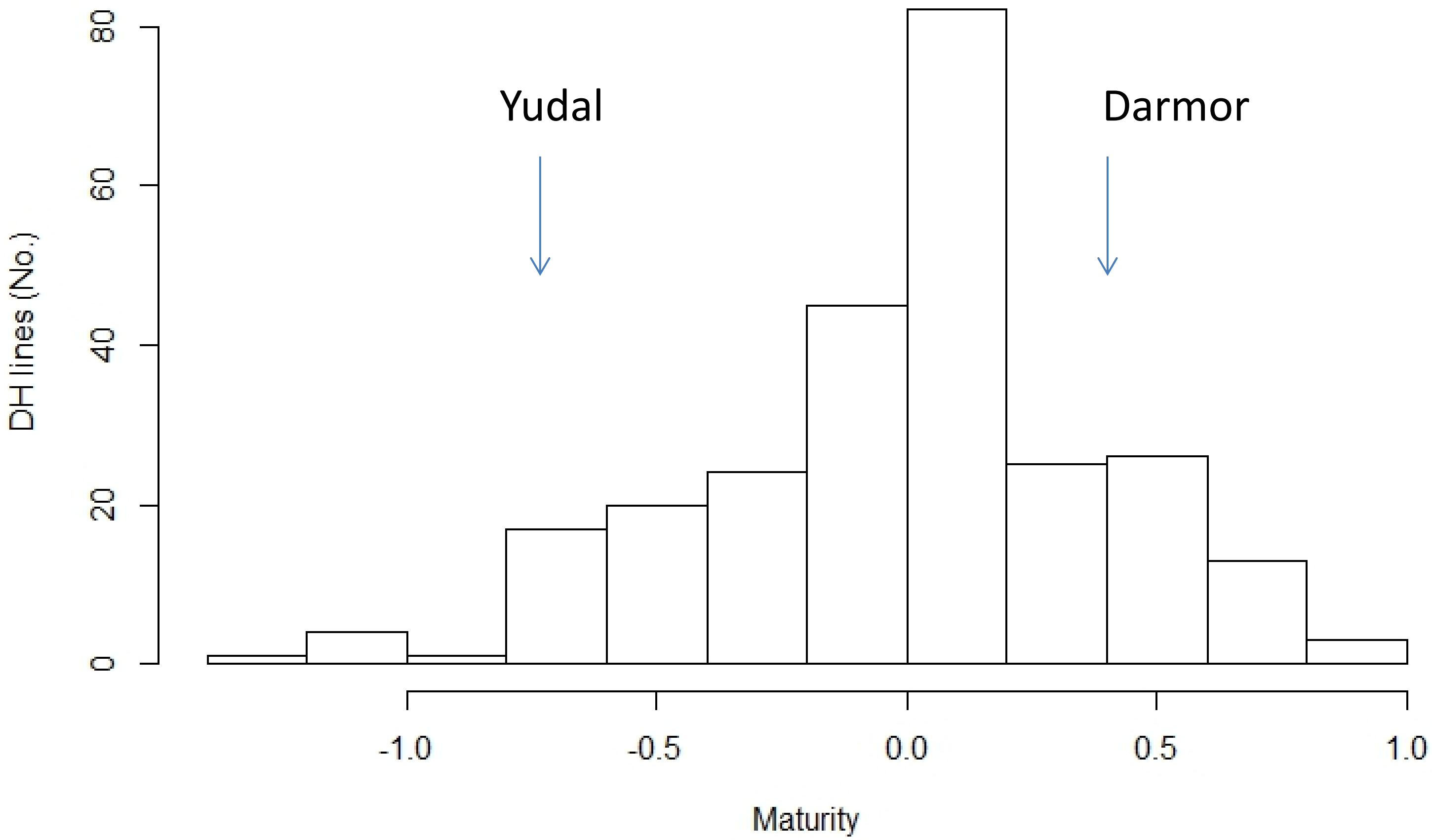

Supplement: Supplementary file 2 [file Image_2.pdf]
