# Supplementary material for: Stable Quantitative Resistance Loci to Blackleg Disease in Canola (Brassica napus L.) Over Continents
Source: Front Plant Sci. 2018 Nov 23;9:1622. doi: 10.3389/fpls.2018.01622 (PMC6265502; doi:10.3389/fpls.2018.01622)
Supplement: Supplementary file 3 [file Image_3.pdf]

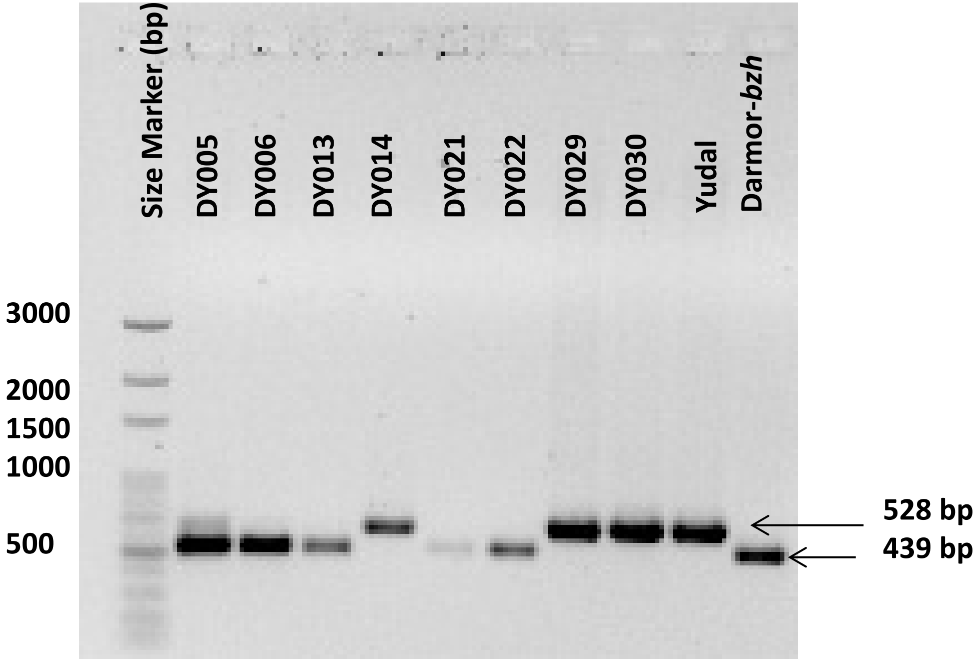

Supplementary Figure 3: Segregation of the *Bzh* gene in selected doubled haploid lines of Darmor-*bzh*/Yudal population. Amplicons were analysed on TAE buffered agarose gel (2.5%) and stained with Syber Green. 100 bp size standard was used for allele size estimation.
