# Supplementary material for: Stable Quantitative Resistance Loci to Blackleg Disease in Canola (Brassica napus L.) Over Continents
Source: Front Plant Sci. 2018 Nov 23;9:1622. doi: 10.3389/fpls.2018.01622 (PMC6265502; doi:10.3389/fpls.2018.01622)
Supplement: Supplementary file 1 [file Image_1.pdf]

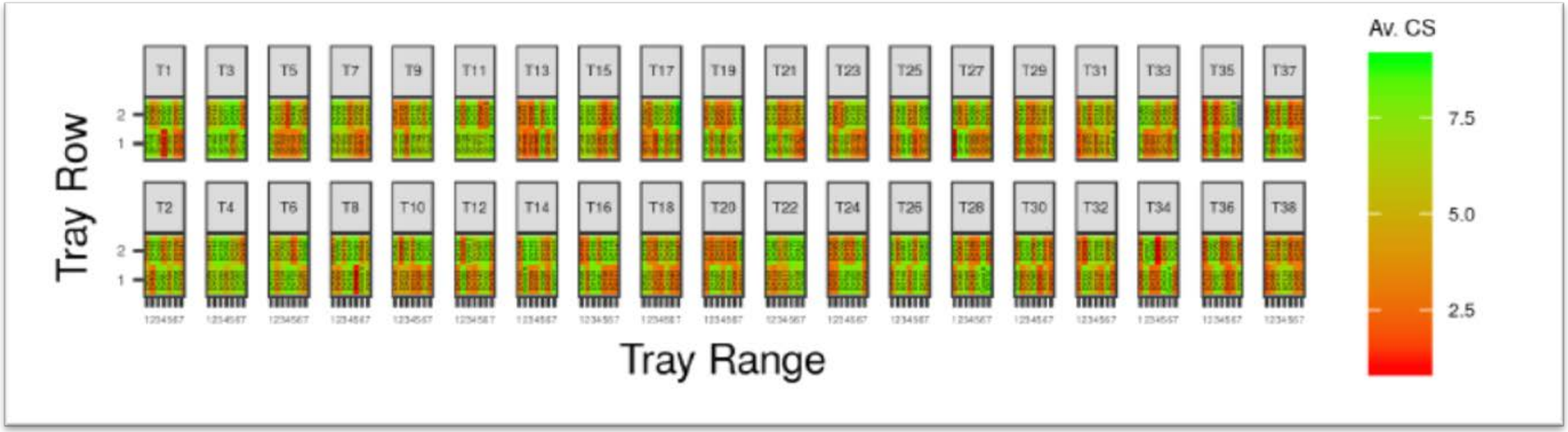

Supplementary Figure 1: Design and heat map of average cotyledon scores for the 2016 glasshouse trial. Note that unlike the representation below there were no physical gaps between cells of neighbouring seedling trays during the actual trial. AV.CS represents to cotyledon scores.
